# Bacterial microcompartments as a next-generation metabolic engineering tool: utilizing nature's solution for confining challenging catabolic pathways

**DOI:** 10.1042/BST20230229

**Published:** 2024-05-30

**Authors:** Lior Doron, Cheryl A. Kerfeld

**Affiliations:** 1 MSU-DOE Plant Research Laboratory, Michigan State University, East Lansing, MI, U.S.A.; 2 Environmental Genomics and Systems Biology and Molecular Biophysics and Integrative Bioimaging Divisions, Lawrence Berkeley National Laboratory, Berkeley, CA, U.S.A.; 3 Department of Biochemistry and Molecular Biology, Michigan State University, East Lansing, MI, U.S.A.

**Keywords:** ancillary proteins, industrially important hosts, nano-bioreactors, non-native multi-enzyme biochemical pathways, synthetic shells, uncapped BMC shells

## Abstract

Advancements in synthetic biology have facilitated the incorporation of heterologous metabolic pathways into various bacterial chassis, leading to the synthesis of targeted bioproducts. However, total output from heterologous production pathways can suffer from low flux, enzyme promiscuity, formation of toxic intermediates, or intermediate loss to competing reactions, which ultimately hinder their full potential. The self-assembling, easy-to-modify, protein-based bacterial microcompartments (BMCs) offer a sophisticated way to overcome these obstacles by acting as an autonomous catalytic module decoupled from the cell's regulatory and metabolic networks. More than a decade of fundamental research on various types of BMCs, particularly structural studies of shells and their self-assembly, the recruitment of enzymes to BMC shell scaffolds, and the involvement of ancillary proteins such as transporters, regulators, and activating enzymes in the integration of BMCs into the cell's metabolism, has significantly moved the field forward. These advances have enabled bioengineers to design synthetic multi-enzyme BMCs to promote ethanol or hydrogen production, increase cellular polyphosphate levels, and convert glycerol to propanediol or formate to pyruvate. These pioneering efforts demonstrate the enormous potential of synthetic BMCs to encapsulate non-native multi-enzyme biochemical pathways for the synthesis of high-value products.

## Introduction

Continuing development of synthetic biology tools has enabled the construction of synthetic metabolic pathways for the production of high-value bioproducts in both well-studied bacterial model organisms and in recently emerged non-model bacterial chassis (reviewed in [1]). However, attempts to redirect metabolic fluxes towards desired products result in unpredictable metabolic bottlenecks or low pathway flux arising from cofactor imbalances, mismatched enzyme kinetics, or accumulation of toxic or unwanted intermediates [2–5]. To cope with these challenges, bioengineers have synthetically achieved spatial organization in prokaryotes by either targeting multiple enzymes to membranous compartments (reviewed in [6,7]), tethering multiple enzymes on protein scaffolds using protein interaction domains, or by encapsulating enzymes in native or synthetic compartments such as virus-like particles, encapsulins, and vault proteins (reviewed in [6,8]) or by using a scaffold-less enzyme organization strategy such as Liquid–Liquid Phase Separation (reviewed in [9]). Spatial organization can also be accomplished by enclosing metabolic pathways within the shell of protein-based prokaryotic organelles known as bacterial microcompartments (BMCs) [10]. BMCs natively encapsulate a segment of a metabolic pathway within a selectively permeable protein shell that acts as a barrier between the encapsulated enzymatic core and the cytosol. They can accommodate a wide diversity of internal enzymatic biochemistries that span from anabolic to catabolic functions [11,12], and provide the bacteria a competitive advantage in specific environmental niches [11,13–15]. To-date, numerous types of BMCs have been experimentally characterized, including the anabolic α- and β-carboxysomes that encapsulate RuBisCO and carbonic anhydrase and act as part of a very efficient carbon-concentrating mechanism in cyanobacteria and some chemoautotrophs [16–19], and the catabolic BMCs (also known as metabolosomes) that assist heterotrophs in the assimilation of diverse organic substrates such as 1,2-propanediol (PDU [20] and GRM3 [21,22]), ethanolamine (EUT [23]), choline (Cut [24] and GRM2 [25,26], small saccharides (GRM5 [27] and PVM [14]), xanthine (XAU [15]), amino alcohols (RMM/AAUM [28]), taurine [29], and aromatic compounds (ARO [30]). The genes that are required for the full functionality of each BMC type are typically clustered in super loci that encode structural proteins that form the BMC shell, its enzymatic core, and ancillary proteins such as regulators, activating enzymes, and transporters that integrate the BMC function into the cell's metabolism [11,12,31]. The understood functional diversity of BMCs is continually expanding with the ever increasing sequencing of the microbial (meta) genomic universe and the ability to use bioinformatic tools to discover the presence of BMCs [32].

**Figure 1.**
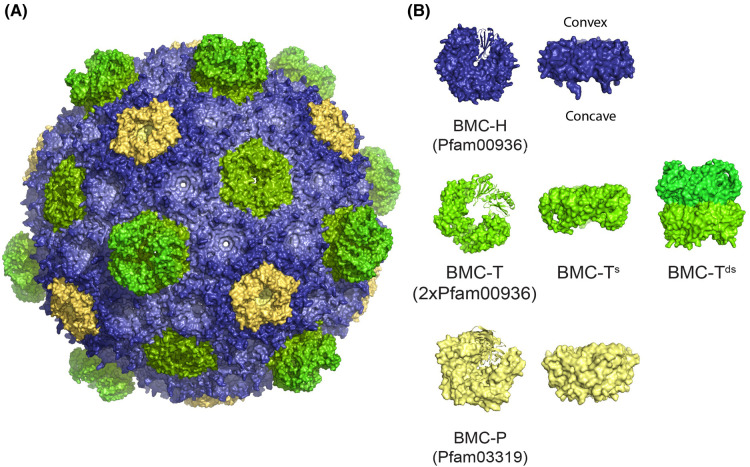
Structural representation of a bacterial microcompartment shell and its shell protein building blocks. (**A**) BMC shells are composed of (**B**) BMC-H (Pfam00936), BMC-T (2xPfam00936) either single (BMC-T^s^) and/or stacked dimers of trimers (BMC-T^D^), and BMC-P (Pfam03319). Natively, the N- and C-termini of hexamers are located on the external surface of the shells.

The functional diversity of BMC is supported by a structurally conserved and modular shell chassis. The BMC shell is typically composed of three types of protein building blocks which, when expressed together, self-assemble into a polyhedral shell (Figure 1A). The shell facets are composed of a monomeric BMC-H protein that contains a unique Pfam00936 domain that forms hexamers [33] and pseudohexamers, BMC-T^s^, which is a genetic fusion of two BMC-H Pfam00936 domains. The pseudohexamers assemble into trimers that resemble the hexamers both in size and shape [34]. In some cases, two trimers dimerize and stack on top of each other (BMC-T^D^) [34,35]. Both BMC-H and BMC-T have a pore formed at the central symmetry axis, which can vary in size and charge to create a channel supporting the selective permeation of metabolites. Lastly, the vertices of the shells are capped by the Pfam03319 domain-containing protein BMC-P, which forms pentamers [36] (Figure 1B). Together, BMC-H, BMC-T, and BMC-P components are the basic building blocks which enable the modular construction of synthetic BMC shells. In the majority of BMCs the targeting of the enzymatic core into the shell interior is mediated via short peptides comprising 15–20 amino acid residues, which are referred to as encapsulation peptides (EPs) [37,38]. EPs are typically found at the C- or N-termini of some BMC enzymes and were predicted [37] and then shown to form amphipathic α-helix structures [39] that facilitated the loading of the enzymatic core through non-covalent interactions with the shell proteins.

The accumulated knowledge of the fundamental principles that drive BMC assembly has reached a tipping point. Thanks to years of structural and functional studies on various types of BMCs, researchers have successfully developed numerous shell models and cargo targeting methods. These developments have enabled the construction of synthetic BMCs with novel functionality geared towards metabolic engineering applications as programmable synthetic nano-bioreactors. Here, we cover the latest developments in synthetic BMC engineering and focus on the potential use of BMC-shell scaffolds as a next-generation metabolic engineering tool to spatially organize metabolic enzymes in bacteria for diverse applications.

## The establishment of synthetic empty BMC shells and BMC-based scaffolds

**Figure 2.**
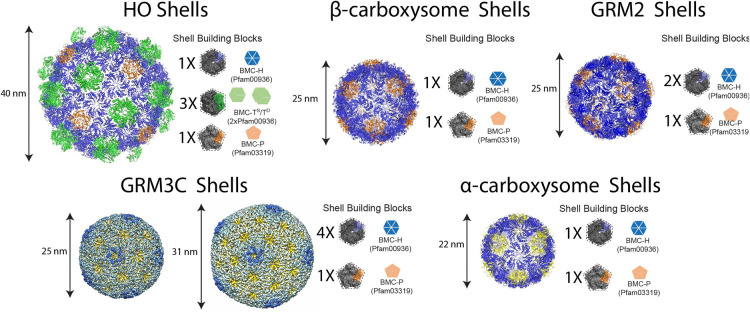
Representations of the structures of synthetic BMC shells determined by crystallography and cryo-EM and their shell protein compositions. BMC shells are composed of BMC-H (Pfam00936), BMC-T (2xPfam00936) either single (BMC-T^s^) and/or dimer stacked (BMC-T^D^), and BMC-P (Pfam03319).

**Figure 3.**
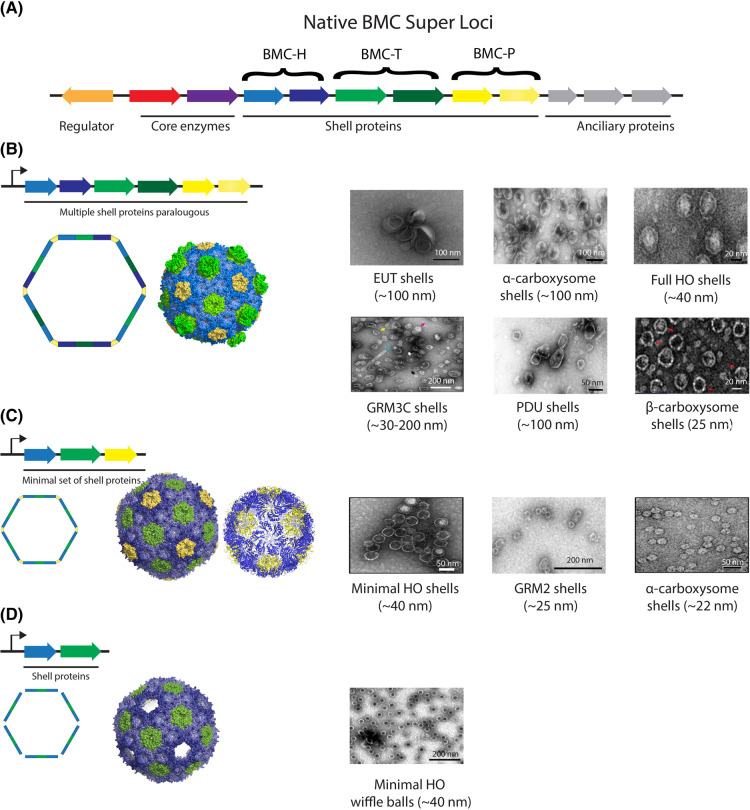
Reducing the complexity of synthetic shells from their native context. (**A**) Overview of the components of a typical native BMC super locus containing a transcriptional regulator (orange), the enzymatic core (purple and red), the structural shell proteins (blue, green, and yellow) forming the BMC shell, and the ancillary proteins positioning and metabolically integrating the BMC into the cell (gray). (Left panel) Diagram of a typical synthetic operons encodes for full (**B**), minimal (**C**), and uncapped shells (**D**) and their observed structures. Different shades of the same color represent shell protein paralogous. (Right panel) Transmission electron microscopy (TEM) micrographs of different negatively stained purified synthetic shell types. TEM Images were obtained from [26,39,41,44,46,49,50].

The development of model BMC systems is a crucial first step towards successfully implementing BMC biotechnology. Such model systems need to be amenable to engineering experiments, easily produced, and can be rapidly purified. To date, seven model shell systems have been established in *Escherichia coli* or structurally characterized by either Cryo-EM or X-ray crystallography. These models include the PDU system [40], EUT system [41], representatives from α- and β-carboxysomes [42–45], GRM2 [26] and GRM3C types [46], and the metabolosome shell derived from the halophilic myxobacterium *Haliangium ochraceum* (HO shells) [47,48] (Figure 2). These efforts demonstrated the ability to form empty shells in the absence of the core enzymes, and ancillary proteins, that are encoded in the main BMC locus (Figure 3A), and to target heterologous proteins to their lumen. Notably, these structural studies demonstrated that the architectural plasticity of recombinantly assembled shell particles varies based on the composition of the shell genes that were expressed (Table 1). In general, the co-expression of multiple paralogous shell proteins can result in the formation of very large and structurally diverse synthetic shells with diameters of 100–200 nm that are morphologically similar to the native microcompartments (Figure 3B). Examples of large microcompartment shell systems include the PDU [40], EUT [41], α-carboxysome [49], and GRM3C [46]. In contrast, the expressions of a single shell protein paralogue from each shell protein type (e.g. one BMC-H, one BMC-T, and one BMC-P), have usually resulted in the assembly of relatively small (25–40 nm) and homogenous icosahedral shells (Figure 3C). This was demonstrated for the GRM2 shells [26], α-carboxysome shells [42,43], β-carboxysome shells [44,45], HO shells [48,50], and for a synthetic BMC-T protein [51]. Despite these examples, the mechanism for controlling shell size is still unclear and could be affected by the presence of the enzymatic core or linker proteins, as was shown in a recent study that demonstrated the involvement of the α-carboxysome linker protein CsoS2 in controlling the shell size [52]. The ability to assemble larger microcompartments might have an advantage when constructing synthetic multi-enzyme BMC-based metabolic pathways because they could provide increased cargo capacity compared with a minimal shell system [10,53]. On the other hand, small shells exhibit a higher surface-to-volume ratio compared with larger compartments (this relationship arises from the equation: $A/V=(4\pi r^{2}/((4/3)\pi r^{3}))$ of a spherical compartment). The increased surface- to-volume ratio in smaller shells would facilitate more efficient diffusion of substrates and products between the core and the exterior of the shell [54], which consequently, may contribute to improved enzyme kinetics within these minimal shells. In addition, using the reductionist shell approach will reduce the complexity of the system, requiring the modification of only one or two shell proteins, compared with the complexity of the full operon that involves multiple paralogues that provide different permeability characteristics to the shell. Interestingly, a recent bioinformatic survey of BMC loci in bacteria has identified several BMC loci with very simple shell protein compositions, which implies that simple shells are also found in nature. These include ARO (two BMC-H and one BMC-P), PVM (two BMC-H and three BMC-P), and two other uncharacterized BMCs; the *Acidobacterium* microcompartment (ACI) and Sugar Phosphate Utilization microcompartment (SPU, specifically SPU4, although a few loci encode a BMC-T gene), that each encode two BMC-H and three BMC-P genes [11].

**Table 1.** The architectural plasticity of recombinantly assembled shell particles based on the shell genes composition

Synthetic shell system	Number of expressed BMC-H genes	Number of expressed BMC-T genes	Number of expressed BMC-P genes	Shell shape	Diameter (nm)	References	Comments
GRM2	2	0	1	Spherical	25	[26]	
α-carboxysome	1	0	1	Spherical	25	[42,43]	
α-carboxysome	3	1	2	Polyhedral shapes	85–100	[49,52]	Include the csoS2 gene
β-carboxysome	1	0	1	Spherical	25	[44,45]	
HO shells	1	1	1	Spherical	40	[48,50]	
HO shells	3	1	1	Spherical	40	[48,50]	
PDU	4	2	1	Polyhedral shapes	100	[40]	
EUT	3	1	1	Polyhedral shapes	100–150	[41]	
GRM3	4	1	1	Polyhedral shapes/nanotubes	30–200	[46]	

In addition to the construction of intact shells *in vivo*, shell assembly can also be accomplished *in vitro.* This was made possible with the development of an assembly method that involves the fusion of a short ubiquitin-like modifier (SUMO) domain to the N-terminus of various shell proteins [55] to inhibit their self-assembling properties and prevent their assembly into supramolecular structures such as sheets [56,57] and nanotubes [40,46,55,58,59]. This allowed for the purification of individual shell proteins, which were used as building blocks in the assembly process. By cleaving the SUMO group from SUMOylated hexamers in the presence of non-tagged trimers and pentamers, the *in vitro* assembly of HO shells, β-carboxysome shells, and engineered BMC-based architectures was demonstrated [55]. To reduce the complexity of the synthetic shell systems even more and eliminate the need to alter shell protein pore residues to adjust permeability, shells can also be formed without pentamers. The uncapped shell architecture (also known as ‘wiffle ball’ shells) [50,51,60], allows the crossing of large metabolites as well as small proteins into and out of the shells through the ∼50 Å gaps at the shell vertices (Figure 3D).

## Adapting shell proteins for bioengineering

**Figure 4.**
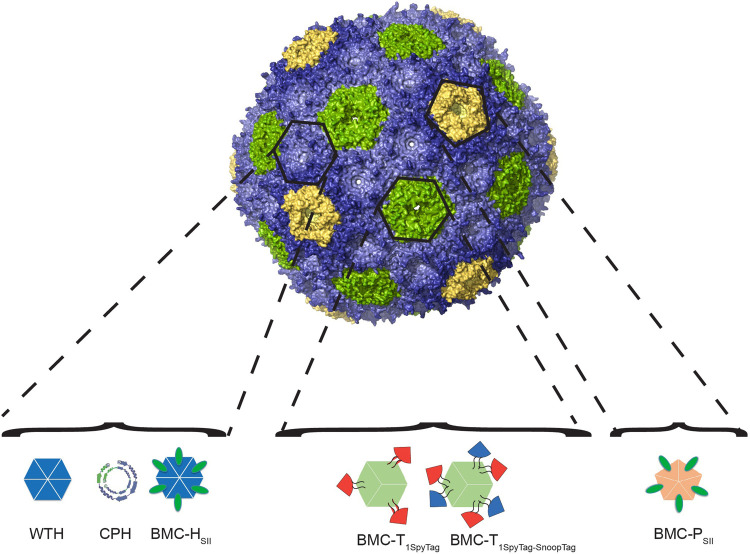
Model of a synthetic shell with functionalized building blocks that were engineered for metabolic engineering purposes. Protein cargo can be genetically fused to the C-terminus of WTH (cargo will be displayed on exterior surface of shell) or CPH (cargo will be encapsulated within the lumen of the shell). SpyTag and SnoopTag split adhesion domains were introduced into a loop within BMC-T_1_ shell proteins to facilitate the encapsulation of cargo proteins. A Strep tag was added to the C-terminus of BMC-P or BMC-H to allow the rapid purification of the loaded shells.

The successful assembly of intact empty shells *in vivo* and the structural characterization of different shell types revealed that the shell protein orientation is conserved across functionally and phylogenetically distinct BMCs, with the N- and C-termini of hexamers projecting out from the external surface of the shells. This guided the direct fusion of protein elements such as affinity tags which facilitated their rapid purification [45,50,55], or lead to the development of synthetic circularly-permuted hexamers (CPHs) with an inverted sidedness of their N- and C-terminal residues relative to their natural counterpart; these display their termini on the luminal surface of the shell. In this case, the direct fusion of protein cargo to CPHs resulted in their encapsulation [61–63]. The structural characterization of different shell types also have provided an atomic resolution blueprint, which guided the incorporation of heterologous domains such as SpyTag/SpyCatcher and SnoopTag/SnoopCatcher split bacterial adhesion domains [64,65] into internal loops of HO BMC-T [50,60], Pdu [66], or CsoS1A [67], to enhance the encapsulation efficiency, which was relatively sparse when heterologous enzymes were fused to EPs [40,47,68–70]. Non-native adhesion systems rely on the formation of a covalent bond through the interaction of the SpyCatcher or SnoopCatcher with the SpyTag or SnoopTag domains, respectively. Notably, the SnoopTag/SnoopCatcher system does not cross-react with SpyTag/SpyCatcher system, and therefore, multiple enzymes can be co-encapsulated at the same time. Furthermore, these two systems, which can be used together with other native and synthetic encapsulation mechanisms to target multiple enzymes to the lumen of shells, offer precise control of loaded enzyme stoichiometry and can be used to control enzymatic cascade activity within microcompartments or on shell protein scaffolds [71,72]. Overall, the development of these encapsulation and display strategies (Figure 4) makes the shells of BMCs an ideal platform for ‘bottom-up’ approaches to construct synthetic BMCs carrying out entirely novel functions.

## The encapsulation of enzymes that catalyze sequential steps in close proximity within BMC shells as a way to prevent the formation of toxic intermediates or mitigate metabolic bottlenecks

In some cases, the ability to engineer microbes to redirect the carbon flux towards the production of high value bioproducts is limited by either the diffusion of pathway intermediates to competing reactions within the cytosol, metabolic bottlenecks in key enzymatic steps in the pathway that hinders the full potential of the pathway, or the toxicity of pathway intermediates [3,5,73,74]. BMC-based spatial organization of heterologous enzymatic pathways represents an elegant approach to minimizing detrimental metabolic interactions within the cytoplasm and enhancing productivity (reviewed in [53]). In addition, BMC-mediated encapsulation offers a way to improve the stability of encapsulated enzymes by their immobilization or their protection from harsh external conditions [75]. BMCs, specifically metabolosomes, have evolved naturally to encapsulate multistep enzymatic processes and to sequester enzymes that form toxic aldehyde compounds or volatile intermediates [11,30,76]. The encapsulation of two or more enzymes that catalyze sequential steps known to produce toxic intermediates in close proximity to each other ensures a quick conversion of the toxic intermediate and enables the utilization of metabolic pathways that are otherwise not accessible due to toxicity.

**Figure 5.**
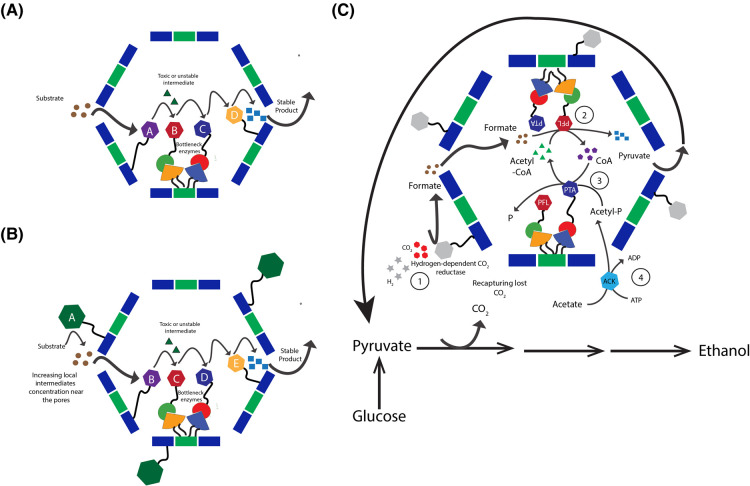
Schematics of possible applications for synthetic BMCs in metabolic engineering. (**A**) Encapsulating multiple metabolic enzymes can prevent toxic intermediate formation or alleviate bottlenecks, enhancing pathway flux. Enzymes can be targeted by fusing to CPHs (**A** and **D**) or utilizing split adhesion bacterial systems (**B** and **C**), ensuring quick conversion of intermediates (green triangle) or boosting catalytic activity. (**B**) The external enzyme (enzyme A) captures cytosolic substrate, converting it into an intermediate to boost local concentration near shell pores. Alternatively, the enzyme can be displayed externally, reducing potential crowding within the lumen by fusing it to wild type hexamer on the external surface of the shell. Enzymes can be targeted externally by fusing to wild type hexamer. (**C**) A possible design to integrate the sFUT into the cell's metabolism. The hydrogen-dependent CO_2_ reductase is displayed on the external surface to recapture the lost CO_2_ (red hexamers) and condense it with H_2_ (gray stars) into formate (1). The concentration of the external formate (brown circle) will increase near the pentamer gaps of the wiffle ball architecture, favoring its diffusion to the interior of the shell to be used by the encapsulated PFL to favor the production of pyruvate (light blue squares) for its recycling (2). CoA (purple petamers) will be recycled back to Acetyl-CoA (green triangles) by the encapsulated PTA, which condenses acetyl with CoA (3). Acetyl phosphate will be supplied by the phosphorylation of Acetate by the non-encapsulated Acetate kinase (4).

Taking advantage of the ability to compartmentalize multiple enzymes near each other in BMC shell scaffolds has allowed bioengineers to construct various novel multienzyme BMC-based metabolic pathways (Table 2). This was demonstrated with the construction of an ethanol nano-bioreactor [39], or a 1,2-propanediol synthesizing BMC module [77] using the PDU microcompartment shell system. These pioneering studies provided evidence for the potential use of synthetic BMCs in the colocalization of multienzyme cascades that would integrate into the cell's metabolism and improve the pathway flux. One potential design, for example, will include the encapsulation of segments of metabolic pathways, especially enzymatic steps that are known to produce problematic intermediates or cause metabolic bottlenecks, to reduce unwanted side reactions or to mitigate the metabolic bottleneck. The formation of intermediates within BMC-based shells could increase local substrate concentration inside the lumen and enhance the catalytic activity of encapsulated enzymes, thereby favoring the biosynthesis of the desired bioproduct (Figure 5A). A possible addition to this synthetic module will be the display of a metabolic enzyme on the external surface of uncapped shells which can be used to produce an intermediate in a high concentration near shell pores that will diffuse to the interior and will be used by the downstream encapsulated enzymes (Figure 5B). Likewise, placement of some enzymes on the external surface help to reduce potential crowding effects within the lumen, which may interfere with the assembly of shells or the activity of the encapsulated enzymes.

**Table 2.** A summary of BMC-based nano bioreactors constructed within the past decade

Name of the developed compartment	Description of the developed BMC	Encapsulation method	Outcome	References
Ethanol nano-bioreactor	Encapsulation of pyruvate decarboxylase and an alcohol dehydrogenase from *Z. mobilis*.	Fusion of native EPs of the PDU system, specifically those of PduP and PduD.	Ethanol production was increased by 63% compared with an unencapsulated control.	[39]
1,2-propanediol synthesizing BMC module	Encapsulation of glycerol dehydrogenase, dihydroxyacetone kinase, methylglyoxal synthase and 1,2-propanediol oxidoreductase.	Fusion of native EPs of the PDU system, specifically those of PduP and PduD.	The four-enzyme pathway resulted in the conversion of the PDU BMC, which is typically involved in the breakdown of 1,2-propanediol, into a 1,2-propanediol synthesizing BMC module, using glycerol as the starting material.	[77]
Hydrogen nano-bioreactor	Encapsulation of an [FeFe]-hydrogenase from *Chlamydomonas reinhardtii* fused to Ferrodoxin and a second ancillary enzyme that catalyzes the transfer of electrons from NADPH to Ferrodoxin.	Fusion of the fused proteins to the C-terminus of CsoS2 that was identified as a native EP for the α-carboxysome shell system.	The encapsulation of the HydA-Fd fusion protein and the ancillary enzyme resulted in 4.1-fold of hydrogen production in aerobically grown *E. coli* cultures compared with cells expressing free HydA-Fd.	[49]
Hydrogen nano-bioreactor	Encapsulation of a [NiFe]-hydrogenases from *E. coli* with its activating enzyme. and a second ancillary enzyme that catalyzes the transfer of electrons from NADPH to Ferrodoxin.			[78]
Synthetic formate-utilizing bacterial microcompartment (sFUT)	Encapsulation of the oxygen-sensitive glycyl radical enzyme, pyruvate formate lyase (PFL), and the acetyl-CoA producing enzyme, phosphotransacetylase (PTA), along with the expression of three other non-encapsulated ancillary proteins PFL-activating enzyme (PFL-AE), S-Adenosylmethionine synthetase (METK), and acetate kinase.	Fusion of SpyCatcher and SnoopCatcher domains to PFL and PTA, respectively.	14 pmol sFUT wiffleballs were able to convert 1 μmol pyruvate to 600 nmol formate.	[60]

## The requirement of ancillary proteins for the construction of novel BMC-based nano-bioreactors

Another aspect that can be useful to consider when designing novel synthetic BMCs is the co-expression of various ancillary proteins such as transporters, regulators, activating enzymes, electron donors or acceptors, or other proteins that are required for the full functionality of the synthetic BMCs [31]. In general, the use of ancillary proteins can aid bioengineers to regulate metabolic pathways, transport a substrate into the cell, efflux a high value product that was generated within the BMC out of the cell, or to situate the synthetic BMC near the cell membrane next to a specific transporter. An example for the use of ancillary proteins was demonstrated in a recent study where the shell of the α-carboxysome, that is thought to be relatively oxygen-impermeable, has been utilized to construct a BMC-based hydrogen nano-bioreactor [49]. The production of hydrogen nano-bioreactor was promoted by encapsulating a fusion of two oxygen-sensitive enzymes, HydA, an [FeFe]-hydrogenase from the green alga *Chlamydomonas reinhardtii*, and the algal Ferredoxin (Fd) that serves as the native electron donor of HydA. This study demonstrated the potential of carboxysome shells in enhancing the catalytic activities of oxygen-sensitive enzymes, due to their O_2_-limited microenvironment. This system was recently improved with the encapsulation of a [NiFe]-hydrogenases from *E. coli* with its activating enzyme. [NiFe]-hydrogenases are relatively O_2_ tolerant and can catalyze H_2_ oxidation in the presence of O_2_ [78]. In another study, the SpyTag/SnoopTag modified HO ‘wiffle ball’ shells with unoccupied pentameric vertices were employed to construct a synthetic formate-utilizing bacterial microcompartment (sFUT). By encapsulating pyruvate formate lyase (PFL), phosphotransacetylase (PTA), and expressing three other non-encapsulated ancillary proteins that are required for the full functionality of the sFUT, the researchers demonstrated the ability of the purified sFUT to convert 1 μmol pyruvate to 600 nmol formate *in vitro* [60]. The expression of the formate transporter FocA, an ancillary protein of the sFUT module, could in the future facilitate the intake of formate from readily available feedstocks, thereby demonstrating the enormous potential of the sFUT prototype to serve as a platform technology in ambitious engineering projects. A possible application would be to utilize the sFUT to mitigate carbon loss when pyruvate is decarboxylated to acetaldehyde during the conversion of lignocellulosic hydrolysates to biofuels [79,80]. A sophisticated strategy to recapture the lost carbon and increase bioproduct's production yield could be to display hydrogen-dependent CO_2_ reductase on the external surface of the uncapped shell. The CO_2_-fixed formate would then diffuse to the BMC interior and converted by the downstream encapsulated enzymes back to a central metabolite, in the case of sFUT, pyruvate (Figure 3C). These examples emphasize the importance and room for further application of ancillary proteins when designing novel synthetic BMCs not only for the functionality of the BMC but also for its ability to integrate it into the cell's metabolism. Future designs, for example, can include the positioning of synthetic BMC-based nano-bioreactors near the cell membrane by the expression of a BMC-H-fused lipid-anchoring protein.

## Introducing BMC shell systems to industrially important hosts

The successful heterologous expression and production of synthetic BMCs in non-native microbial hosts such as *E. coli*, hold substantial promise within the realm of industrial biotechnology. The use of *E. coli* as a model bacterial host for newly designed BMCs has many advantages. First, its rapid, inexpensive growth and high transformation efficiency allows for the examination of the functionality of the modified encapsulated metabolic enzymes that often require many design–build–test cycles or the functionality of the synthetic BMCs. Second, *E. coli* can use a wide variety of substrates for growth ranging from glucose to waste products [81], which could be used as initial substrates for the enzymes encapsulated inside the synthetic BMCs to test their activity *in vivo*. Third, its high expression level of proteins can result in a high yield of the desired bioproduct that is being produced within the microcompartment. However, in some cases, the use of *E.coli* may be suboptimal for the production of the desired bioproduct due to an incompatible metabolic network or its intolerance to extreme conditions. Therefore, the introduction of BMC-based spatial organization into industrial bacterial hosts and other non-model microorganisms is an attractive approach, especially if the metabolic pathways that are being encapsulated are associated with the formation of toxic intermediates or dead-end products that could interfere with the metabolism of the new bacterial host. In an effort to pave the way towards expanding the use of BMC systems to other industrially important bacterial hosts, several studies have introduced BMC shell systems into non-model organisms. The first two studies attempted to achieve a BMC-based spatial organization in *Corynebacterium glutamicum*, an established industrial workhorse for the production of amino acids by introducing either the full gene cluster of the α-carboxysome from *Halothiobacillus neapolitanus* [82], or the shell components of the PDU system from *Citrobacter freundii* [68]. To facilitate proper assembly of the PDU shells in *C. glutamicum*, the researchers optimized protein stoichiometry by modifying the start codon of several shell genes from ATG to GTG and demonstrated that a reduction in the levels of PduK was a key step to successful PDU compartment assembly in *C. glutamicum*. Heterologous expression of the PDU system from *Salmonella* was also examined in various Gram negative bacteria to examine which hosts might be compatible with production of the PDU BMC [83]. To execute their heterologous expression studies, the authors cloned a 38 kb region coding the PDU and cob/cbi cobalamin biosynthetic genes into a plasmid with a broad host range across Gram negative bacteria and attempted the expression and purification of BMCs from these strains. The study provided a foundation for BMC use in a variety of bacterial species using a full, intact clone. In another study, BMC shell assembly and incorporation of a heterologous enzymatic cargo has been achieved in the biotechnologically relevant Gram-positive model organism, *Bacillus subtilis* [84]. To do that, the genes encoding the shell proteins of the PDU BMC from the thermophile *Parageobacillus thermoglucosidasius* were cloned into a synthetic operon and introduced into the chromosome of *B. subtilis*. Assembly of a BMC in *B. subtilis* offers the opportunity to generate novel multienzyme pathways BMC-based nano-bioreactors in this industrial-relevant organism. In a recent study, the HO shell system was introduced into the industrially significant microbe *Zymomonas mobilis* (*Z. mobilis*), thereby establishing a BMC-based spatial organization in *Z. mobilis* for the future production of valuable chemicals [85]. In this study, the authors successfully expressed and purified pentamer capped and uncapped shells loaded with protein cargo from *Z. mobilis*, as well as demonstrated the HO shells’ ability to simultaneously encapsulate and externally decorate proteins of interest on the HO BMC shell scaffolds. The ability to control the orientation of the cargo enzymes will allow the future design of synthetic BMCs for spatial metabolic engineering. For example, it may be possible to simultaneously display and encapsulate rate-limiting enzymes of the methylerythritol phosphate (MEP) pathway to mitigate the diffusive loss of MEP pathway intermediates and improve the carbon flux of the pathway towards the production of isopentenyl diphosphate (IDP) and its isomer dimethylallyl diphosphate (DMADP), known isoprenoid precursor molecules [86,87]. Isoprenoids are a large and diverse group of natural compounds [88,89], which have commercial applications as pharmaceuticals, agrochemicals, pigments, and fragrances [90]. A possible target for encapsulation would be the rate-limiting Fe–S-containing enzyme 4-hydroxy-3-methyl-butenyl 1-diphosphate reductase (also known as IspH), that catalyzes the last step of the MEP pathway resulting in the formation of IDP and DMADP. The displaying of the IspH upstream partner, 2-C-methyl-d-erythritol-2,4-cyclodiphosphate reductase (also known as IspG), on the external surface of the shells could increase IspH substrate concentration near the pores, leading to enhanced activity of the encapsulated enzyme [91]. Overall, these studies exemplify the robustness of the BMC shell platform and highlight their promise for the production of high-valued products in these newly BMC-introduced bacterial hosts.

## Conclusions

The ability to construct capped and uncapped BMC shells and to target multiple enzymes to their interior and exterior surfaces positions BMC-based compartmentalization as an advanced next-generation tool in metabolic engineering. Bioengineers can now design BMC-localized synthetic metabolic pathways to produce desired bioproducts that function independently of the cell's regulatory and metabolic networks and introduce them to both model and non-model bacterial hosts. Further studies need to be carried out to explore the engineering of shell pores to allow the selective transport of reaction specific small molecules. This can be done by mutating specific amino acids in the shell proteins [92], introducing transport proteins that selectively bind and facilitate the transport of specific molecules, or creating hybrid BMCs by combining shell proteins from different BMC systems [93,94]. In addition, the utilization of other BMC-based architectures such as nanotubes [40,59,95] and sheets [47] should also be considered for metabolic engineering applications. This will require the development of nanotubes-forming CPHs and the incorporation of adhesion domains such as SpyTag or SnoopTag to these building blocks to allow the targeting of metabolic enzymes to their interior surface. Alternatively, the direct fusion of cargo proteins to nanotube-forming proteins will allow the utilization of nanotubes as protein scaffolds.

## Perspectives

- The utilization of engineered BMC shells as scaffolds or compartments for metabolic enzymes holds the promise of enhancing product yields in metabolic engineering, while also paving the way for novel possibilities in biotechnology and synthetic biology.
- Bioengineers can now design BMC-localized synthetic metabolic pathways for the production of desired bioproducts that function independently of the cell's regulatory and metabolic networks and to introduce them to both model and non-model bacterial hosts.
- Further studies should be carried out to test the ability of BMC-encapsulated multi-enzyme pathways to integrate into the cell's metabolism, to enhance a native pathway flux, or to carry out the biosynthesis of bioproducts on an industrial scale.

## Abbreviations

BMC: bacterial microcompartment
CPH: circularly-permuted hexamer
DMADP: dimethylallyl diphosphate
EP: encapsulation peptide
MEP: methylerythritol phosphate
PFL: pyruvate formate lyase
PTA: phosphotransacetylase
SUMO: short ubiquitin-like modifier
